# A novel heterozygous frameshift pathogenic variant in *GCM2* gene causing isolated hypoparathyroidism: a case report

**DOI:** 10.3389/fendo.2025.1589182

**Published:** 2025-06-27

**Authors:** Ayano Onishi, Yoshinari Obata, Tomoaki Hayakawa, Makoto Fujiwara, Yasuhisa Ohata, Yuri Tamura, Satoshi Kawata, Kosuke Mukai, Kazuyuki Miyashita, Kenichi Yamamoto, Takuo Kubota, Atsunori Fukuhara, Iichiro Shimomura

**Affiliations:** ^1^ Department of Metabolic Medicine, Graduate School of Medicine, The University of Osaka, Yamadaoka, Suita, Osaka, Japan; ^2^ Department of Pediatrics, The University of Osaka Graduate School of Medicine, Yamadaoka, Suita, Osaka, Japan; ^3^ Laboratory of Children’s Health and Genetics, Division of Health Sciences, The University of Osaka Graduate School of Medicine, Yamadaoka, Suita, Osaka, Japan; ^4^ Department of Genetic Counseling, The University of Osaka Hospital, Yamadaoka, Suita, Osaka, Japan; ^5^ Department of Adipose Management, Graduate School of Medicine, The University of Osaka, Yamadaoka, Suita, Osaka, Japan

**Keywords:** hypoparathyroidism, GCM2, heterozygous, novel variant, case report

## Abstract

Glial cells missing transcription factor 2 (*GCM2*) is one of the genes responsible for isolated hypoparathyroidism. Most cases of hypoparathyroidism caused by *GCM2* pathogenic variants result from homozygous or compound heterozygous loss-of-function variants, with only a limited number of heterozygous variants reported. A 24-year-old woman with recurrent tonic convulsions was admitted to our hospital. Laboratory findings revealed severe hypocalcemia (1.28 mmol/L), normophosphatemia (1.36 mmol/L), and low intact parathyroid hormone levels (0.84 pmol/L). Based on this, hypoparathyroidism was diagnosed. Comprehensive gene analysis using next-generation sequencing revealed a novel heterozygous frameshift pathogenic variant (c.1366delG, p.Ala456ProfsTer75) in the *GCM2* gene (NM_004752.4). Sanger sequencing of the patient and parents confirmed *de novo* occurrence. This variant is predicted to exert a dominant-negative effect by impairing *GCM2* function. This case provides further evidence that heterozygous *GCM2* variants can lead to hypoparathyroidism. Additionally, it underscores the importance of genetic testing for hypoparathyroidism of unknown etiology even in adults.

## Introduction

Hypoparathyroidism is a rare endocrine disorder characterized by hypocalcemia and hyperphosphatemia caused by insufficient parathyroid hormone (PTH). It has several causes, such as neck surgery, radiotherapy, autoimmune disorders, or hypomagnesemia (1, 2). Genetic abnormalities are rare causes of hypoparathyroidism and are generally categorized into syndromic forms, such as 22q11.2 deletion syndrome, and a non-syndromic form, namely isolated hypoparathyroidism (3, 4). Several genes associated with the development or function of the parathyroid gland, including *PTH* and the calcium-sensing receptor (*CASR)*, have been implicated in the pathogenesis of isolated hypoparathyroidism, which can follow autosomal dominant, autosomal recessive, or X-linked inheritance patterns (3, 4). One of the genes responsible for isolated hypoparathyroidism is glial cells missing transcription factor 2 (*GCM2*), which is located on chromosome 6p24.2 (3–5). *GCM2* is comprised of five exons and encodes a zinc finger-type transcription factor consisting of 506 amino acids (5). This protein includes a DNA-binding domain, transactivation domain 1, an inhibitory domain, and transactivation domain 2 (TAD2), which are organized sequentially from the N-terminal region (5, 6). *GCM2* plays a critical role in the development and maintenance of the parathyroid gland (7–10), and several pathogenic variants associated with isolated hypoparathyroidism have been identified. Most cases of hypoparathyroidism caused by *GCM2* pathogenic variants result from homozygous or compound heterozygous loss-of-function variants, with a limited number of heterozygous variants reported (11–14). Herein, we present the case of a patient diagnosed with isolated hypoparathyroidism with a novel *de novo* heterozygous frameshift pathogenic variant of the *GCM2* gene.

## Case presentation

A 24-year-old woman was diagnosed with hypoparathyroidism and admitted to our hospital. She was born full term with no perinatal or infantile developmental abnormalities. At 12 years of age, QT prolongation was observed on an electrocardiogram during a medical checkup; however, further examination was not performed. Since 19 years of age, she has experienced recurrent painful muscle spasms in cold weather and numbness in the extremities following episodes of diarrhea. At 22 years of age, she often experienced difficulty in moving because of numbness and rigidity, although these symptoms resolved spontaneously within a short time. At 24 years of age, she was urgently admitted to a local hospital with repeated tonic convulsions following a viral infection. Laboratory findings revealed severe hypocalcemia (1.28 mmol/L [ref: 2.15–2.57 mmol/L]), normophosphatemia (1.36 mmol/L [ref: 0.94–1.55 mmol/L]), mild hypomagnesemia (0.62 mmol/L [ref: 0.74–0.99 mmol/L]), and a low intact PTH level (0.84 pmol/L [ref: 2.72–7.97 pmol/L]). The patient’s renal function was normal (estimated glomerular filtration rate, 85.1 mL/min/1.73 m^2^). She was diagnosed with primary hypoparathyroidism and started on oral and intravenous electrolyte supplementation. Subsequently, she was transferred to our hospital for further evaluation and treatment.

**Figure 1:**
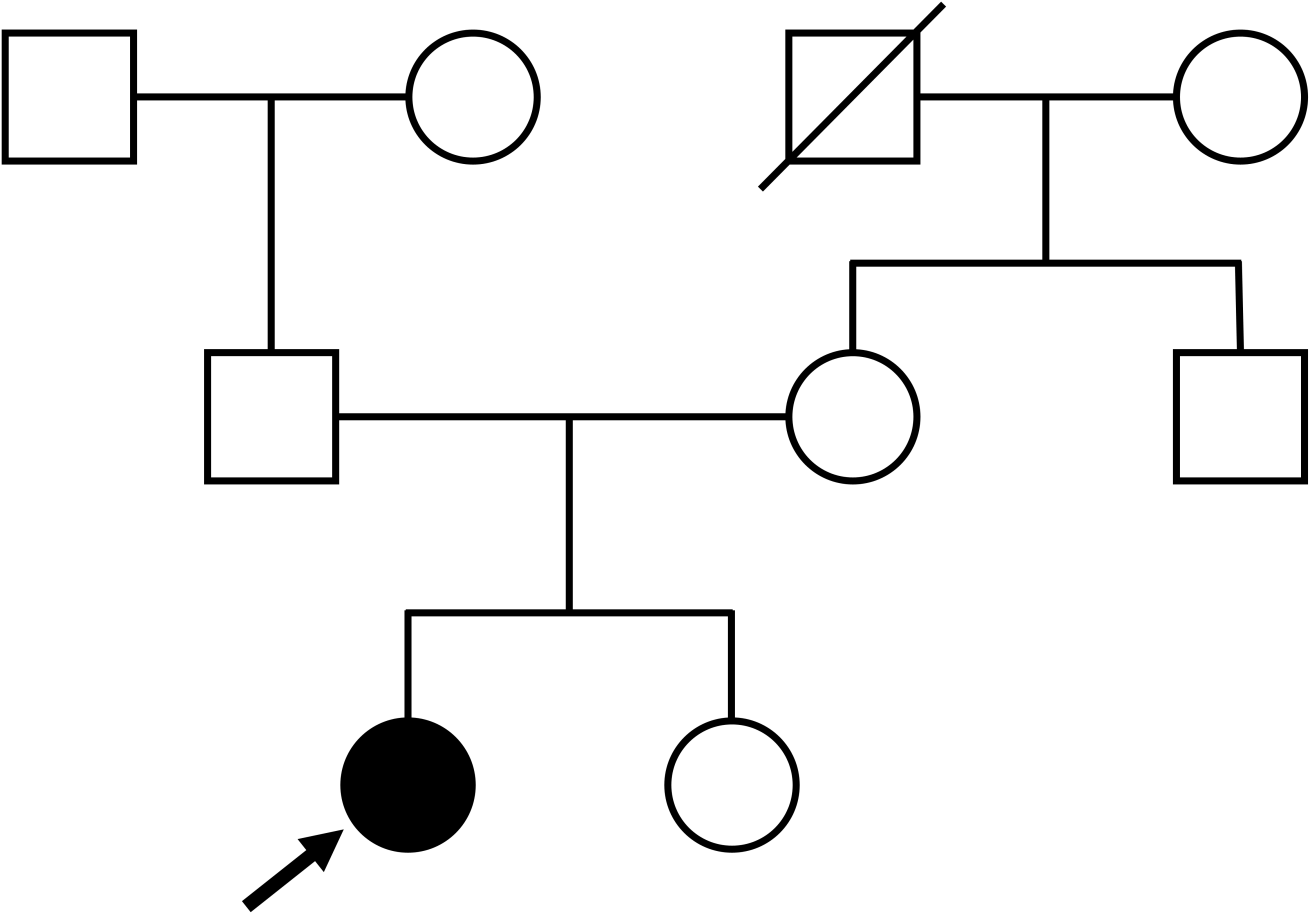
Pedigree diagram of the patient. Arrow indicates the patient.

**Figure 2:**
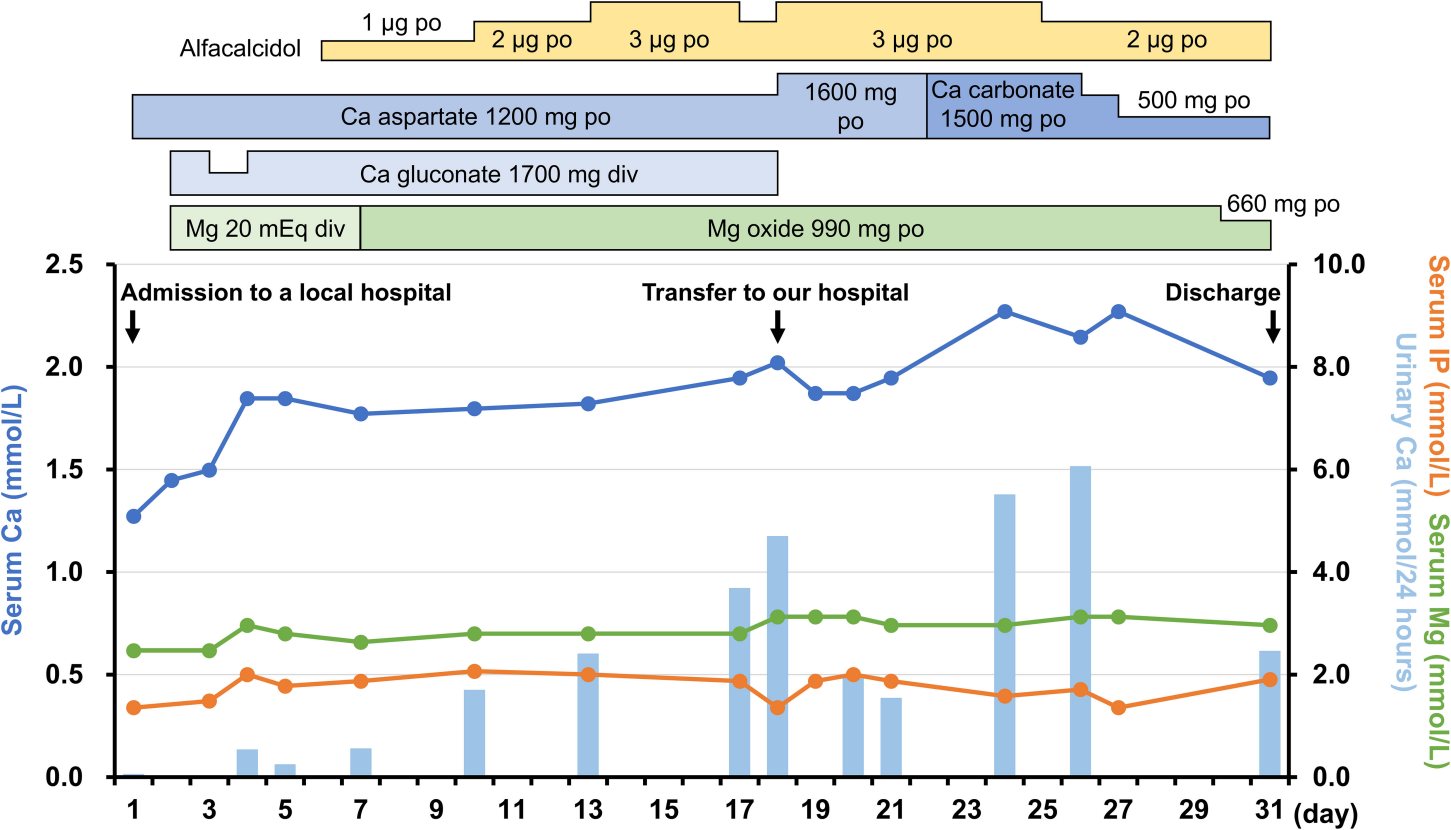
Trends in treatment and laboratory data from the previous hospital admission to discharge at our hospital. The 24-hour urinary calcium excretion was estimated using spot urine samples based on urinary creatinine levels. Ca, calcium; IP, phosphorus; Mg, magnesium.

On admission, the patient’s height, weight, and body mass index were 149.0 cm, 58.0 kg, and 26.1 kg/m^2^, respectively. She had no medical history of neck surgery or autoimmune disease and had not consumed alcohol. The pedigree diagram is shown in **Figure 1**. The patient had one younger sister. She had no family history of calcium metabolism or other endocrine disorders. Physical examination revealed no abnormal findings, such as abnormal facial appearance or hearing loss. Computed tomography (CT) of the head revealed ectopic calcifications in the bilateral caudate nucleus, putamen, globus pallidus, and cerebellar dentate nucleus. Abdominal CT showed no renal malformations or nephrocalcinosis. Bone mineral density was normal (T-score: 1.2 at the lumbar spine, 1.9 at the femoral neck). Hypocalcemia persisted despite the normalization of serum magnesium levels, eliminating hypomagnesemia as the cause of hypoparathyroidism. Although her 1,25-dihydroxyvitamin D levels were within the reference range (62.4 pmol/L [ref: 48.0–144.0 pmol/L]), low 25-hydroxyvitamin D (42.2 nmol/L [ref: >74.9 mmol/L]) was indicative that vitamin D deficiency may have worsened the hypocalcemia. Trends in treatment and laboratory data are presented in **Figure 2**. We continued to supplement the patient with calcium, magnesium, and vitamin D, which resulted in appropriate serum calcium levels. She was discharged with calcium carbonate 500 mg/day, magnesium oxide 660 mg/day, and alfacalcidol 2 μg/day. Alfacalcidol was chosen as the vitamin D analog because of its longer half-life and less frequent dosing, which improved adherence compared with that by calcitriol. Following further adjustments to her supplementation regimen, her serum calcium levels remained stable near the lower limit of the reference range without hyperphosphatemia, hypercalciuria, renal impairment, or symptom recurrence.

**Figure 3:**
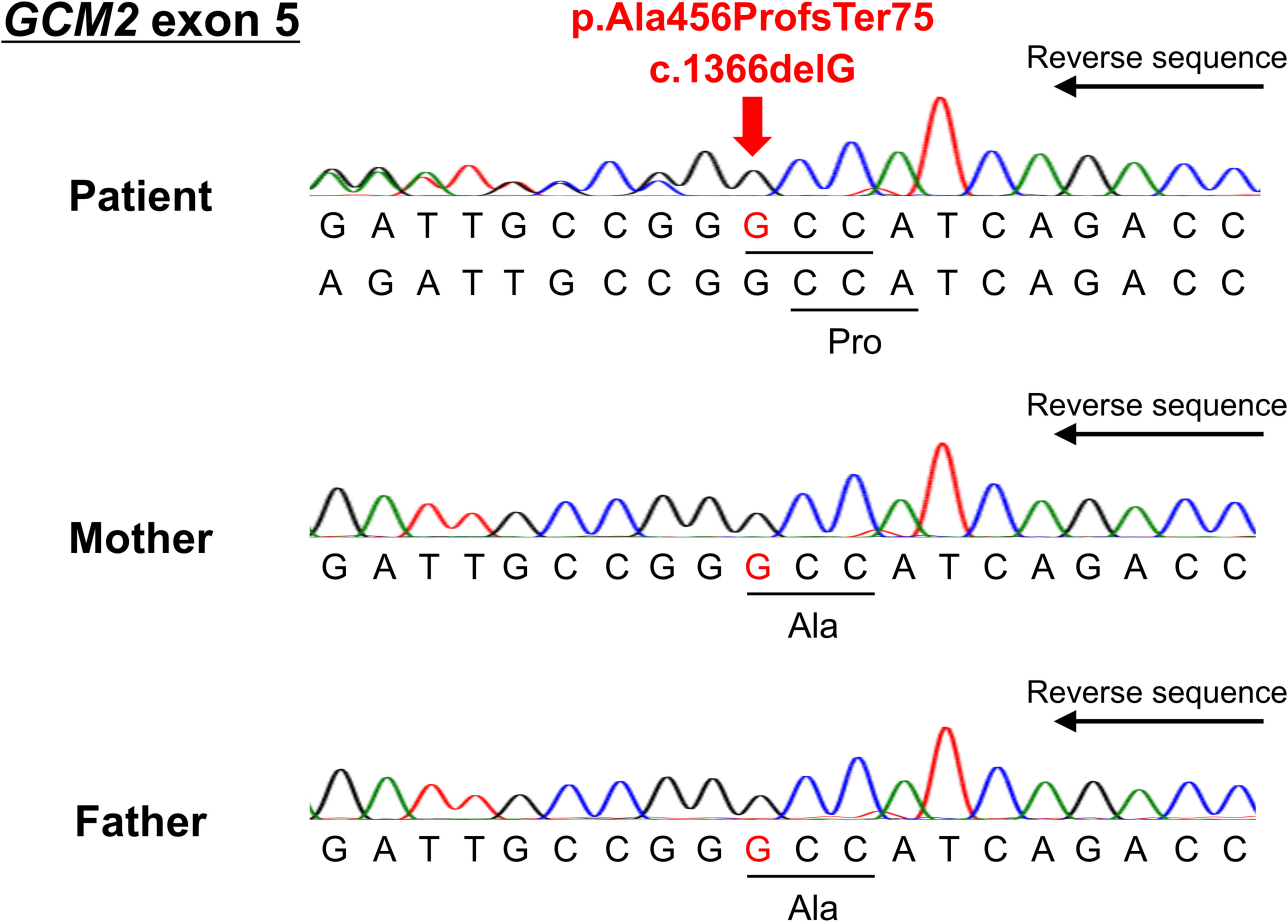
Reverse sequencing results of exon 5 of the *GCM2* gene for the patient and her parents. The deletion of guanine at position 1366 resulted in a frameshift after amino acid 456 (arrow).

Although she had no family history of hypoparathyroidism or syndromic features, the presence of QT prolongation and muscle symptoms during her youth suggests that genetic abnormalities may have contributed to the hypoparathyroidism. The coding and splicing regions of 22 major genes associated with calcium metabolism disorders were analyzed using the NextSeq Sequencing System (Illumina, San Diego, CA, USA) at the Kazusa DNA Research Institute (Kisarazu, Japan). The genes analyzed included *CASR, GNA11, GCM2, TBX1, TBX2, NEBL, CHD7, SEMA3E, GATA3, TBCE, FAM111A, PTH, SOX3, AIRE, NLRP5, HADHA, HADHB, ACADM, DHCR7, CLDN16, CLDN19*, and *TRPM6*. As a result, we identified a novel heterozygous frameshift variant (c.1366delG, p.Ala456ProfsTer75) of the *GCM2* gene (NM_004752.4). The variant was located within the TAD2 region of exon 5 and caused a frameshift, replacing the C-terminal end of the putative TAD2 with 75 unrelated amino acid residues. This variant has not been previously reported in the literature, ClinVar, or the Genome Aggregation Database. Sanger sequencing confirmed the variant in the patient, but it was absent in the parents, who exhibited normal calcium levels, indicating that it was *de novo* (**Figure 3**). According to the American College of Medical Genetics and Genomics guidelines (15), this heterozygous *GCM2* frameshift variant was classified as pathogenic (PVS1+PS2+PM2) and determined to be the cause of hypoparathyroidism in this patient.

## Discussion

Herein, we present the case of a woman diagnosed with hypoparathyroidism. Although the patient had no family history of the disease or other features suggestive of genetic abnormalities, genetic testing revealed a novel *de novo* heterozygous frameshift pathogenic variant of *GCM2* that contributed to the development of hypoparathyroidism.

In 1999, *GCM2* was first identified in humans as an ortholog of the Drosophila glial cells missing gene (5). In mammals, *GCM2* is specifically expressed in developing and mature PTH-secreting cells of the parathyroid glands, and *Gcm2* knockout mice lack parathyroid glands (7). Additionally, conditional *Gcm2* knockout in adult mice inhibited cell proliferation, increased cell death in the parathyroid glands, and caused hypoparathyroidism (8). *GCM2* contributes to the maintenance of parathyroid function by regulating *CASR* and *PTH* gene expression (9, 10). These findings highlight that *GCM2* plays a critical role in the development and maintenance of the parathyroid glands.

**Figure 4:**
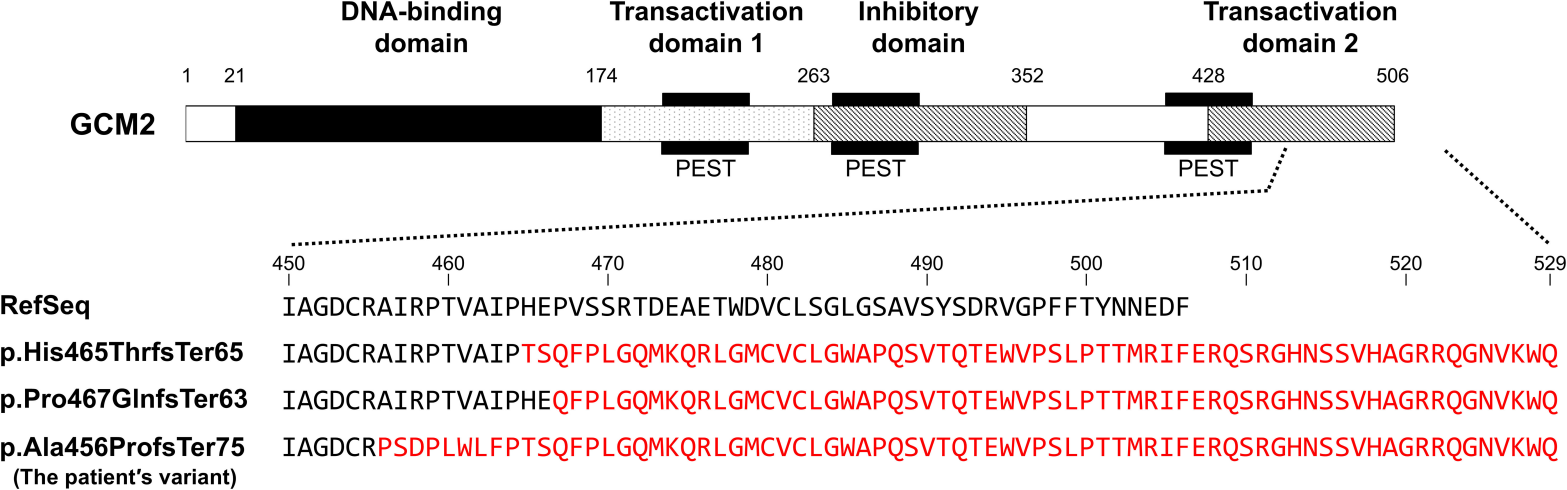
A schematic diagram of the GCM2 protein and comparison of GCM2 amino acid sequences, including the reference sequence (RefSeq), previously reported frameshift pathogenic variants (p.His465ThrfsTer65 [reference: (22, 23)] and p.Pro467GlnfsTer63 [reference (22):]) with confirmed dominant-negative effects and the patient’s variant (p.Ala456ProfsTer75). Mutated amino acids are highlighted in red.

Hypoparathyroidism caused by *GCM2* pathogenic variants was first reported in 2001 (11). Thereafter, several pathogenic variants were identified with no specific variant hotspots. Most reported cases involve homozygous or compound heterozygous loss-of-function variants of *GCM2*, leading to autosomal recessive inheritance (11–14). This observation is consistent with findings in heterozygous *Gcm2* knockout mice, which exhibit a normal phenotype (7), suggesting that simple haploinsufficiency may not be sufficient to cause disease. In contrast, seven heterozygous *GCM2* variants associated with hypoparathyroidism have been reported to date (**Table 1**) (16–23). Since no parent-specific genetic imprinting has been reported for the *GCM2* gene, heterozygous variants must exert a dominant-negative effect to cause the disease. However, two heterozygous missense variants in the DNA-binding domain (c.316T>C [p.Cys106Arg] and c.328C>T [p.Arg110Trp]) have not been shown to exert dominant-negative effects in *in vitro* assays (16–18). Similarly, two other heterozygous variants (c.737dupA [p.Asp246GlufsTer25] and c.1460C>T [p.Ser487Phe]) have yet to be evaluated for these effects (19, 20). Therefore, these four variants may require additional alterations in another allele (compound heterozygosity) or other genes (digenic/polygenic inheritance) to manifest a pathogenic phenotype, although haploinsufficiency cannot be completely ruled out. Consequently, only three heterozygous variants have been confirmed to cause hypoparathyroidism via dominant-negative effects, as demonstrated by luciferase reporter assays (**Table 1**) (21–23). All three pathogenic variants were located in the TAD2 region, which is critical for *GCM2* transcriptional activity. Two of these variants are frameshifts that replace approximately 50% of the C-terminus of putative TAD2 with unrelated amino acid residues, similar to the variant identified in the present patient (**Figure 4**) (22, 23). Although the variant in our patient was not evaluated using an *in vitro* assay, its high similarity to previously reported pathogenic variants strongly suggests that the variant protein exerts a dominant-negative effect, impairing *GCM2* function and leading to hypoparathyroidism. The absence of this variant in the patient’s normocalcemic parents and the lack of abnormalities in other candidate genes further support the pathogenicity of this heterozygous variant.

**Table 1:** Summary of reports on heterozygous *GCM2* variants associated with hypoparathyroidism.

	Nucleotide	Protein	Exon	Domain	First reported year	Dominant-negative effect
Yi et al. (16), Park et al. (17)	c.316T>C	p.Cys106Arg	2	DBD	2012	Not confirmed
Tomar et al. (18)	c.328C>T	p.Arg110Trp	2	DBD	2010	Not confirmed
Singhania et al. (19)	c.737dupA	p.Asp246GlufsTer25	5	TAD1	2022	NA
García-Castaño et al. (20)	c.1460C>T	p.Ser487Phe	5	TAD2	2021	NA
Mirczuk et al. (21)	c.1504A>C	p.Asn502His	5	TAD2	2010	Confirmed
Mannstadt et al. (22), Canaff et al. (23)	c.1389delT	p.His465ThrfsTer65	5	TAD2	2008	Confirmed
Mannstadt et al. (22)	c.1399delC	p.Pro467GlnfsTer63	5	TAD2	2008	Confirmed
Present report	c.1366delG	p.Ala456ProfsTer75	5	TAD2	2025	NA

DBD, DNA-binding domain; TAD1, transcriptional activation domain 1; TAD2, transcriptional activation domain 2; NA, not applicable

In contrast, gain-of-function *GCM2* variants have been reported to cause autosomal dominant hyperparathyroidism 4 (24), suggesting that distinct *GCM2* variants can lead to opposing phenotypes via different molecular mechanisms. This underscores the critical role of *GCM2* in regulating parathyroid function and the importance of understanding how different variants affect its activity.

Most patients with hypoparathyroidism without a family history, diagnosed during adulthood, may not undergo genetic testing in clinical practice. However, patients with isolated hypoparathyroidism exhibit a wide spectrum of clinical features, ranging from asymptomatic mild hypocalcemia to symptomatic severe hypocalcemia, even among individuals with the same pathogenic variant (4). As a result, while many cases of isolated hypoparathyroidism are diagnosed in early childhood owing to typical symptoms, some cases may remain undiagnosed until adulthood because of mild or nonspecific symptoms (4). Consequently, genetic abnormalities may have been under-diagnosed in some adult patients classified as having “idiopathic” hypoparathyroidism. Currently, there are no standardized criteria for patient selection or preferred methodologies for genetic testing. However, precise genetic testing is essential not only to identify the underlying cause of unknown etiology, but also to provide appropriate genetic counseling, particularly for patients of reproductive age. Therefore, genetic testing should be considered in patients with hypoparathyroidism of unknown etiology, including adults. Furthermore, a comprehensive analysis of the responsible genes using next-generation sequencing, as was performed in the present patient, may be especially valuable for uncovering the genetic causes of the disease.

This study has certain limitations. First, we did not assess whether the variant in our patient exerts a dominant-negative effect using an *in vitro* assay. Second, we cannot entirely rule out the presence of large deletions or structural variations in another allele or other genes, which may be challenging to detect using next-generation sequencing.

In summary, we report a case of isolated hypoparathyroidism in an adult woman, caused by a novel *de novo* heterozygous frameshift pathogenic variant of the *GCM2* gene. This case provides further evidence that heterozygous *GCM2* variants can lead to hypoparathyroidism. Additionally, it underscores the importance of genetic testing for hypoparathyroidism of unknown etiology even in adults.

## Data Availability

The datasets presented in this article are not publicly available due to privacy restrictions. Requests to access the datasets should be directed to the corresponding author.
